# *Brevibacillus brevis* HNCS-1: a biocontrol bacterium against tea plant diseases

**DOI:** 10.3389/fmicb.2023.1198747

**Published:** 2023-09-13

**Authors:** Wenbo Yang, Hui Yang, Xiaocun Bao, Mehboob Hussain, Qiang Bao, Zexuan Zeng, Chun Xiao, Lingyun Zhou, Xiaoping Qin

**Affiliations:** ^1^College of Plant Protection, Yunnan Agricultural University, Kunming, China; ^2^Tea Research Institute, Hunan Academy of Agricultural Sciences, Changsha, China

**Keywords:** *Brevibacillus brevis*, antagonistic activity, genome annotation, pan-genome analysis, antimicrobial peptides, edeine

## Abstract

As a biocontrol bacteria, *Brevibacillus* has been the subject of extensive research for agricultural applications. Antibacterial peptides (AMPs) are the main antibacterial products of *Brevibacillus*. This study isolated a strain of *Br. brevis* HNCS-1 from tea garden soil, and the strain has an antagonistic effect against five types of pathogens of tea diseases, namely *Gloeosporium theae-sinensis*, *Elsinoe leucospila, Phyllosticta theaefolia, Fusarium sp., and Cercospora theae*. To determine the genetic characteristics implicated in the biocontrol mechanism, the genome sequence of the HNCS-1 strain was obtained and analyzed further, and the data are deposited in the GenBank repository (No. CP128411). Comparative genomics analyses revealed that the HNCS-1 strain and 17 public *Br. brevis* share a core genome composed of 3,742 genes. Interestingly, only one non-ribosomal peptide synthetase (NRPS) gene cluster annotated as edeine is present in the core genome. And UHPLC-MS/MS detection results showed that edeine B and edeine A were the principal antibacterial peptides in the HNCS-1 strain. This study proves that edeine is the main antibacterial peptide of *Br. brevis*, and provides a new strategy for the identification of antibacterial products from other biocontrol bacteria.

## Introduction

The tea plant (*Camellia sinensis*) is an important cash crop, and its beneficial metabolites are valuable for human health (Pan et al., 2022). Similar to other plants, tea plants are vulnerable to many diseases throughout their life cycle. Among them, fungal diseases are the most serious hazards to tea plants, resulting in a decline in tea production and quality. For example, tea white scab disease occurs year-round in the Chinese tea host production area, generally resulting in a reduction of approximately 10% in production and a reduction of over 50% in the sick tea garden (Zhou et al., 2019). Tea gray blight can reduce tea production by 10–20% (Sanjay et al., 2008). For a long time, chemical control has been the main means to address tea plant diseases. However, there are fewer pesticides registered for tea plant diseases, and long-term single and excessive use of chemical pesticides has caused problems such as pathogen resistance, pesticide residues, and environmental pollution. Therefore, tea plantations urgently need a sustainable, environmentally friendly, and anti-drug-resistance plant disease control technology to replace traditional chemical control. Microbial pesticides have become one of the ideal strategies for the comprehensive control of tea plant diseases due to their persistence and environmental friendliness.

*Brevibacillus* spp. is an essential source of biocontrol microorganisms and is widespread in nature, having been discovered in soil, flora, seawater, and the intestinal tracts of animals (Ruiu, 2013). There are 20 species in the genus, including 10 *Bacillus* species discovered previously (the *Br. brevis* cluster). *Br. brevis* is the parent species of the genus *Brevibacillus* (Shida et al., 1996). It has been reported in the disciplines of biological control of plant maladies, pollution degradation, and heavy metal remediation (Samrot et al., 2015; Che et al., 2016; Wang et al., 2016). *Brevibacillus* is one of the most studied bacterial groups, and it has been one of the most important producers of antimicrobial peptides (AMPs) (Yang and Yousef, 2018). AMPs play an important role in bacteriostasis (Xu et al., 2023). AMPs can be classified according to their biosynthetic pathways as ribosomally synthesized and post-translationally modified peptides (Ripps) and non-ribosomally synthesized peptides (NRPS). Currently, the preponderance of *Brevibacillus* AMPs are produced by non-ribosomal peptide synthetases. They consist of edeine (A, B, D, and F) (Czajgucki et al., 2006), gramicidin (A-C, S) (Govaerts et al., 2001; Kessler et al., 2004), gratisin (Tamaki et al., 1983), tyrocidine (A-D) (Mootz and Marahiel, 1997), BT1583 (Wu et al., 2005), tostadin (Song et al., 2012), tauramamide (Desjardine et al., 2007), brevistin (Shoji and Kato, 1976), spergualin (Takeuchi et al., 1981), loloatin (A-D) (Gerard et al., 1999), laterocidin (Xu et al., 2010), and tridecapeptide families (Yang et al., 2017). *Brevibacillus* spp. generate numerous Ripps, such as laterosporulin (Singh et al., 2012), laterosporulin10 (Baindara et al., 2016), and Bac-GM100 (Ghadbane et al., 2013). Most *Brevibacillus* AMPs exert their antimicrobial effect *via* cytoplasmic membrane damage, but edeine inhibits DNA synthesis and protein translation and synthesis at varying concentrations (Szer and Kurylo-Borowska, 1972; Dinos et al., 2004).

However, the diversity of types and structures of AMPs has also led to difficulties in the purification and identification of traditional chemical analysis techniques. Fortunately, the development of next-generation sequencing technology over the past two decades has consequently stimulated research on the comparative genomics of *Br. brevis*. More biocontrol strains of *Br. brevis* have had their entire genomes sequenced in order to investigate the relationships between antibacterial mechanisms and the underlying genetic diversity of *Br. brevis* genomes. With the advancement of bioinformatics prediction, AMP gene clusters have been continuously identified. The metabolic pathway database of AMPs, which can annotate the protein gene clusters of biocontrol bacteria at the gene level and predict the results of secondary metabolites, has been perpetually improved, making the rapid identification of AMPs feasible. In addition, with the continuous publication of multiple microbial genomes and the comparison of microbial genomes between different individuals of the same species, it is gradually recognized that a single reference genome cannot represent diversity within a species (Cuellar-Gaviria et al., 2023; Wan et al., 2023).

In this study, a strain of *Br. brevis* HNCS-1 with broad-spectrum antibacterial activity was isolated from the tea garden soil. To gain a comprehensive understanding of the biocontrol potential of strain HNCS-1, we sequenced the genome of *Br. brevis* HNCS-1. A comparative genomic analysis was conducted to characterize the pan-genome structure of this biocontrol bacterium, assembled with 17 public *Br. brevis* genomes. An NRPS gene cluster was found in the core genome, which may be related to the antibacterial ability of *Br. brevis*. This study provides a genetic context for future research on AMPs and provides a scientific basis for further optimizing the field applications of the microbial biopesticide derived from *Br. brevis* HNCS-1.

## Materials and methods

### Microbial culture and preservation

The cultivation and plant protection laboratory of the Tea Research Institute of the Hunan Academy of Agricultural Sciences isolated *Br. brevis* HNCS-1 from tea garden soil and deposited it in China Center for Type Culture Collection (No. M 2022713). The cultivation and plant protection laboratory isolated and preserved *Gloeosporium theae-sinensis, Elsinoe leucospira, Phyllosticta theaefolia, Fusarium* sp., and *Cercospora theae*.

The fungi were routinely cultivated at 26°C on a PDA medium (6 g potato extract, 20 g dextrose, 20 g agar, 1,000 ml H_2_O, pH 5.6 ± 0.2) and stored at 4°C on the same medium. Before using them in an experiment, they were activated on a PDA medium for 24 h at 26°C and then transferred by streaking. The HNCS-1 strain was stored in 20% glycerol at −80°C. It was activated by streaking at 37°C for 12 h on LB agar medium (10 g tryptone, 5 g yeast extract, 10 g NaCl, 15 g agar, 1,000 ml H_2_O, pH 7.0 ± 0.1).

### Antibiotics activity of *Brevibacillus brevis* HNCS-1

*Br. brevis* HNCS-1 was inoculated into 500 ml Erlenmeyer flasks containing 200 ml of the LB liquid medium and cultivated at 37 °C and 200 rpm for 12 h. Then, 10 ml of the seed cultures were transferred to 500 ml Erlenmeyer flasks containing the NB medium (10 g peptone, 3 g beef extract, 5 g NaCl, 1,000 ml H_2_O, and pH 7.2 ± 0.2) and cultivated at 26 °C and 180 rpm for 3 days. *Br. brevis* HNCS-1 fermentation broth was centrifuged at 4°C and 10,000 rpm for 10 min, and the supernatant was collected and filtered through a 0.22 μm filter.

On a PDA medium containing a 10% cell-free supernatant of *Br. brevis* HNCS-1, spore germination was used to determine the bacteriostatic activity against fungi. A fungus cake was made using a 5 mm diameter sterile cork borer and placed in the center of the plate. Plates of *P. theaefolia, Fusarium* sp., *E. leucospila, C. theae*, and *G. theae-sinensis* were incubated at 26 °C for 12 days, 11 days, 22 days, 22 days, and 7 days, respectively. The experiment was performed thrice. The antagonistic activity of *Br. brevis* HNCS-1 was evaluated by inhibition rate (IR), which is calculated using the following formula:


$$\begin{array}{l} IR\left(\%\right)=\frac{\left(A-5\right)-\left(B-5\right)}{A-5}\;\times \;100 \end{array}$$

where *A* is the diameter of tea fungus disks in the control treatment, *B* is the diameter of tea fungus disks with *Br. brevis* HNCS-1, and 5 is the diameter of the disks inoculated with tea fungus.

### Genome sequencing, annotation, and alignment

The entire genome of *Br. brevis* HNCS-1 was sequenced using the Illumina and Nanopore platforms. Guangdong Magigene Technology Co., Ltd. performed this study in China. Following the standard protocol provided by Oxford Nanopore Technologies (ONT), the sequencing procedure includes sample quality detection, library construction, library quality detection, and library sequencing. SMRT Link v5.1.0 was chosen for the assembly of third-generation data alone, and Unicycle was chosen for the assembly of second-generation and third-generation data. tRNAscan-SE v1.3.1, rRNAmmer v1.2, and Rfam databases briefly predicted Glimmer, tRNAs, and rRNAs, respectively. PHAST identified prophage sequences within the genome assemblies. Island viewer predicted Genomic Islands (GIs).

The complete nucleotide sequence was also searched against Non-Redundant (NR) Protein Database, Swiss-Port, Cluster of Orthologous Groups (COGs), Kyoto Encyclopedia of Genes and Genomes (KEGG), Gene Ontology (GO), Pfam, Carbohydrate-Active enZYmes (CAZy) database, Pathogen–Host Interactions (PHIs), Virulence Factor Database (VFDB), and Comprehensive Antibiotic Resistance Database (CARD) for functional annotation and further function assignment. Basic Local Alignment Search Tool (BLAST) and Diamond were used to compare the gene sequence with the reference database sequences. The finest matching results were selected based on identity, e-value, and score, which were the results of genome annotation. Circular maps of the genome were generated using Circos (Krzywinski et al., 2009).

### Phylogenetic tree and pan-genome analysis

*Br. brevis* HNCS-1 and 17 other *Br. brevis* genomes were used to construct a phylogenetic tree, with *B. subtilis* ATCC 13952 serving as an out-group. Except for *Br. brevis* HNCS-1, all sequences were obtained from the National Center for Biotechnology Information (NCBI) (Table 1). OrthoFinder software (Emms and Kelly, 2019) was used to generate a maximum likelihood (ML) phylogenetic tree, which was visualized using Figtree v1.4.4. To evaluate the phylogeny of the *Br. brevis* strains, average nucleotide identity (ANI) was calculated using the FastANI software (Jain et al., 2018). The thermal map was then visualized using the TBtools software (Chen et al., 2020).

**Table 1:** Genome features of *Brevibacillus brevis* strains used in this study.

**Strain**	**Assembly**	**Size (Mb)**	**GC%**	**Level**	**CDS**	**Source**	**Country**
Ag35	GCA_014526365.1	6.48	47.3	Contig	5808	Root nodule	America
ATCC 35690	GCA_002161835.1	6.13	47.3	Contig	5567	Soil	Poland
B011	GCA_022026395.1	6.16	47.5	Complete	5552	Tobacco roots	China
DSM 30	GCA_003385915.1	6.61	47.4	Scaffold	6224	-	-
DZQ7	GCA_001039275.2	6.44	47.4	Complete	5825	Tobacco rhizosphere soil	China
FJAT-0809-GLX	GCA_000346255.1	6.02	47.3	Contig	5527	Rhizosphere soil of watermelon	China
G25-137	GCA_015912885.1	6.34	47.1	Scaffold	5801	-	China
GZDF3.1	GCA_001649505.1	6.43	46.8	Scaffold	5924		
HK544	GCA_007725005.1	6.49	47.3	Complete	5892	Soil	South Korea
I2-B3	GCA_019749035.1	6.22	47.4	Contig	5615	Air filter	America
LABIM17	GCA_021401445.1	5.95	47.5	Chromosome	5445	Soil from rainforest	Brazil
NBRC 100599	GCA_000010165.1	6.30	47.3	Complete	5776	-	-
NBRC 110488	GCA_001748185.1	6.28	47.3	Scaffold	5847	-	-
NBRC 15304	GCA_006539845.1	6.52	47.4	Contig	6152	-	-
NCTC2611	GCA_900637055.1	6.73	47.4	Complete	6241	-	-
NRRL NRS-604	GCA_003012835.1	6.61	47.4	Contig	6208	Soil	America
X23	GCA_000296715.2	6.64	46.9	Complete	6197	Soil of vegetable field	China

To identify the core and strain-specific genes, a pan-genome analysis was performed on 18 *Br. brevis* isolates using the BPGA v1.3 software, which implemented functional ortholog clustering using the amino acid sequences based on Gene Family (GF) approach (Chaudhari et al., 2016). The collection of genes shared by all 18 strains was referred to as the pan-genome, while the set of shared genes within the test genomes was referred to as their core genomes. For pan-genome genetic contexts, Heap's Law can be represented by the following formula:


$$\begin{array}{l} y=k\times n^{\gamma } \end{array}$$

where *y* represents the extent of the pan-genome, n represents the number of genomes, and k and γ are fitting parameters. According to this law, γ can be calculated as α = 1–γ, so when α < 1 (0 < γ < 1), the extent of the pan-genome increases unboundedly with the successive addition of new genomes and is considered open. In contrast, if α > 1 (γ < 0), the pan-genome trajectory approaches a plateau and can be considered closed as additional genomes are added (De Jesus et al., 2022).

### Prediction of genes related to antibacterial activity

The NR database was chosen as the protein sequence database for our annotation system based on a balance of quality, comprehensiveness, and our practical needs. Using the complete nucleotide sequence of the *Br. brevis* HNCS-1 genome, the online tools antiSMASH 6.0 (Blin et al., 2021) were able to identify clusters of secondary metabolite genes.

### Isolation and antibiotic activity of AMPs

One thousand milliliters of *Br. brevis* HNCS-1 supernatant was frozen at −80°C for 24 h and then concentrated to 20 ml by vacuum freeze drying. The concentrated sample (2 ml) was separated by column chromatography on Sephadex G-75 using an H_2_O-CH_3_OH gradient [H_2_O, H_2_O-CH_3_OH = 4:1 (v/v), H_2_O-CH_3_OH = 3:2 (v/v), H_2_O-CH_3_OH = 2:3 (v/v), H_2_O-CH_3_OH = 1:4 (v/v), CH_3_OH]. The eluent was collected using an EP tube, ranging from 0.7 ml to 1.0 ml per tube. The cell-free eluate's antibacterial activity against *G. thea-sinensis* was determined using agar diffusion on a PDA medium after it was filtered through a 0.22 μm filter membrane. In total, 1 ml of *G. thea-sinensis* spore suspension was distributed across the agar surface of the plates. Then, a sterilized cork borer was used to create 5 mm diameter wells with equal spacing in the agar. Overall, 3 days were spent incubating the dishes at 26°C. The experiment was performed thrice.

### UHPLC-MS/MS method

For the separation, ultra-high-performance liquid chromatography (Exion, SCIEX) was utilized. The process utilized an Agilent ZORBAX SB-C18 column (2.1 mm × 150 mm, 2.7 μm) with acetonitrile (A) and 0.1% formic acid in water (B) as gradient elution mobile phases at a flow rate of 0.3 ml/min. The gradient elution protocol was as follows: 5% B from 0–5 min, 5–50% B from 5–20 min, 50–100% B from 20–30 min, and 100% B from 30–35 min. The temperature of the column was set to 35°C.

The X500R Q-TOF mass spectrometer (SCIEX, Framingham, MA, USA) was used to acquire untargeted mass spectral data. The LC effluent was injected into the mass spectrometer. ESI parameters were as follows: temperature: 500°C; ion source gas 1 and 2: 50 psi; curtain gas: 35 psi; CAD gas: 7 psi. Collision-induced dissociation at 35 ± 15 V in IDA mode was utilized to capture the MS and MS/MS spectra. Maximum candidate ions: 10; threshold for intensity: 600 cps; full scan mass range: 500–1,000 Da; ion discharge voltage: 5,500 V; collision energy: 35 V; collision energy spread: 15 V.

## Results

### Biocontrol activity of *Brevibacillus brevis* HNCS-1

**Figure 1:**
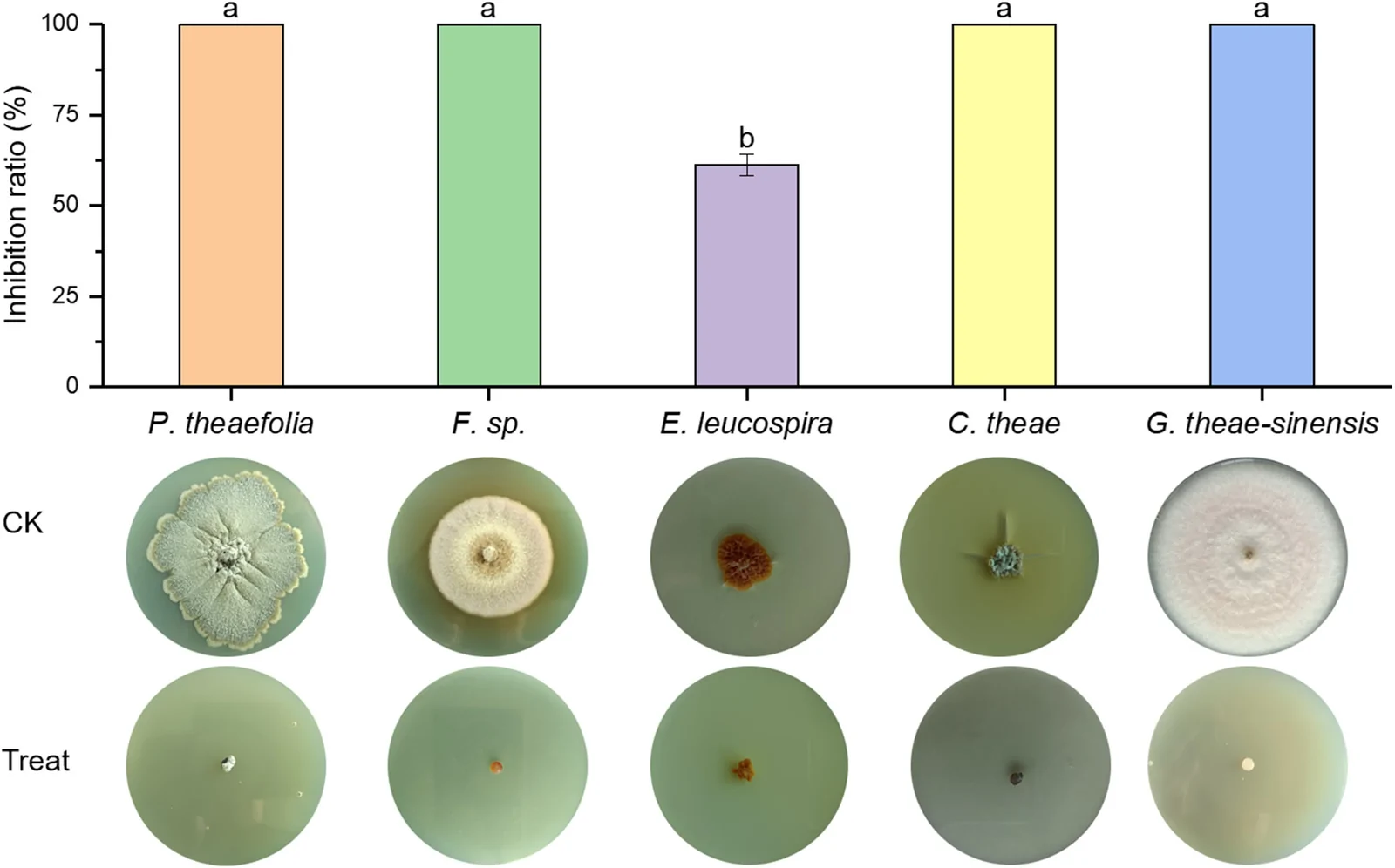
Antimicrobial activity of 10% *Brevibacillus brevis* HNCS-1 supernatant against five tea fungal diseases. The five tea fungal diseases from left to right are *Phyllosticta theaefolia, Fusarium* sp., *Elsinoe leucospira, Cercospora theae*, and *Gloeosporium theae-sinensis*. Different lowercase letters on the bar chart indicate significant differences in data at the 0.05 level.

*Br. brevis* HNCS-1 was evaluated for its antifungal activity against pathogenic fungi. The results demonstrated that HNCS-1 inhibited the mycelia proliferation of five tea fungal diseases significantly (Figure 1). *Br. brevis* HNCS-1 was isolated from tea garden soil and demonstrated broad-spectrum antagonistic activity. The mycelial growth of *Phyllosticta theaefolia, Fusarium* sp., *Cercospora theae*, and *Gloeosporium theae-sinensis* was completely inhibited by 10% *Br. brevis* HNCS-1 supernatant. Additionally, it significantly inhibits the mycelial proliferation of *Elsinoe leucospira*. *Br. brevis* HNCS-1 is a beneficial microbe with biotechnological application potential.

### General genome description of *Brevibacillus brevis* HNCS-1

The genome of the HNCS-1 strain consists of a single circular chromosome measuring 6,353,630 bp with a GC content of 47.15%; no plasmids were detected (GenBank CP128411). This chromosome genome consisted of 6,342 coding DNA sequences (CDS), which accounted for 87.50% of the genome. In addition, 15 sRNA, 44 rRNA, and 127 tRNA were predicted based on the chromosome sequence. In the genome, 31 Genomic Islands were identified, but no CRISPR repeat regions or prophages were found (Supplementary Figure 1).

In total, 5,796 and 4,080 identified genes were annotated as NR and Swiss-port, respectively, and 2,916 and 4,714 genes were classified into functional categories based on GO and COG designations, respectively (Supplementary Figures 2, 3). Overall, 5,810 KEGG pathways have been assigned (Supplementary Figure 4). Moreover, 4,808, 2,906, 1,190, 173, and 6 genes, respectively, were annotated in Pfam, PHI, VFDB, CAZyme, and CARD (Supplementary Figures 5, 6).

### Whole-genome phylogenetic analysis of *Brevibacillus brevis*

**Figure 2:**
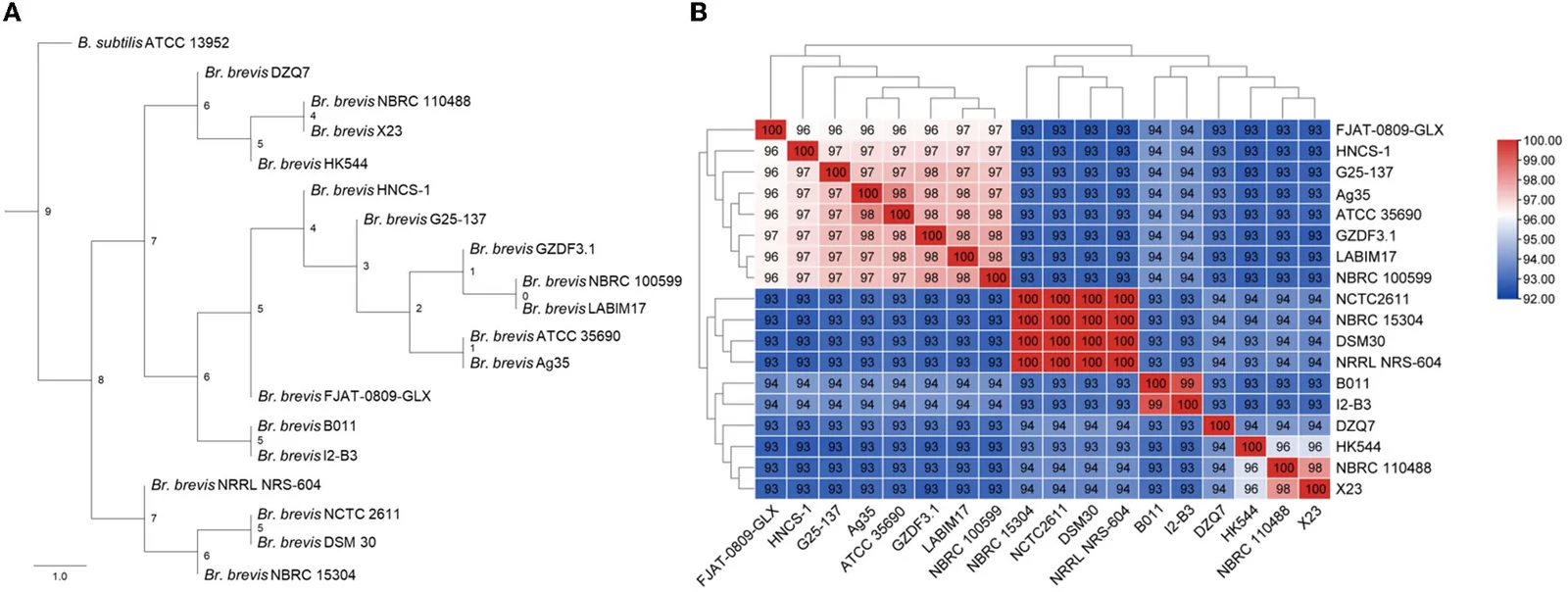
Phylogenetic tree of *Brevibacillus brevis*. **(A)** Maximum likelihood tree of different *Br. brevis* by 18 genomes and an out group (*Bacillus subtilis* ATCC 13952). **(B)** Heat map of average nucleotide identity values among different strains of *Br. brevis* revealing five groups.

The phylogenetic tree of 18 *Br. brevis* genomes and *B. subtilis* ATCC 13952 was constructed using the ML method and all orthogroups, with the ATCC 13952 strain serving as the root. It was found that HNCS-1, *Br. brevis* strains 17, and ATCC 13952 formed a large cluster, and different strains of *Br. brevis* revealed two groups (Figure 2A). In the meantime, strain HNCS-1 was a member of the same clade as strains G25-137, Ag35, ATCC 35690, GZDF3.1, NBRC100599, and LABIM17, but it was not a sister group to any other strains.

To substantiate the results of the phylogenetic analysis, we also determined the ANI values of various strains. Strains with ANI values above 95% are regarded as belonging to the same species (Richter and Rossello-Mora, 2009). The results showed that the genetic relationship between ANI and phylogenetic trees was basically consistent, but ANI was more precise, dividing 18 strains of *Br. brevis* into 5 species (Figure 2B).

According to the results, they were not distinguished by the mixed trend of geographical origin and phylogenetic clustering of strains isolated from similar environments. The genome's phylogenetic clustering differed substantially from the strains' specific habitat classification.

### The pan-genome features of *Brevibacillus brevis*

**Figure 3:**
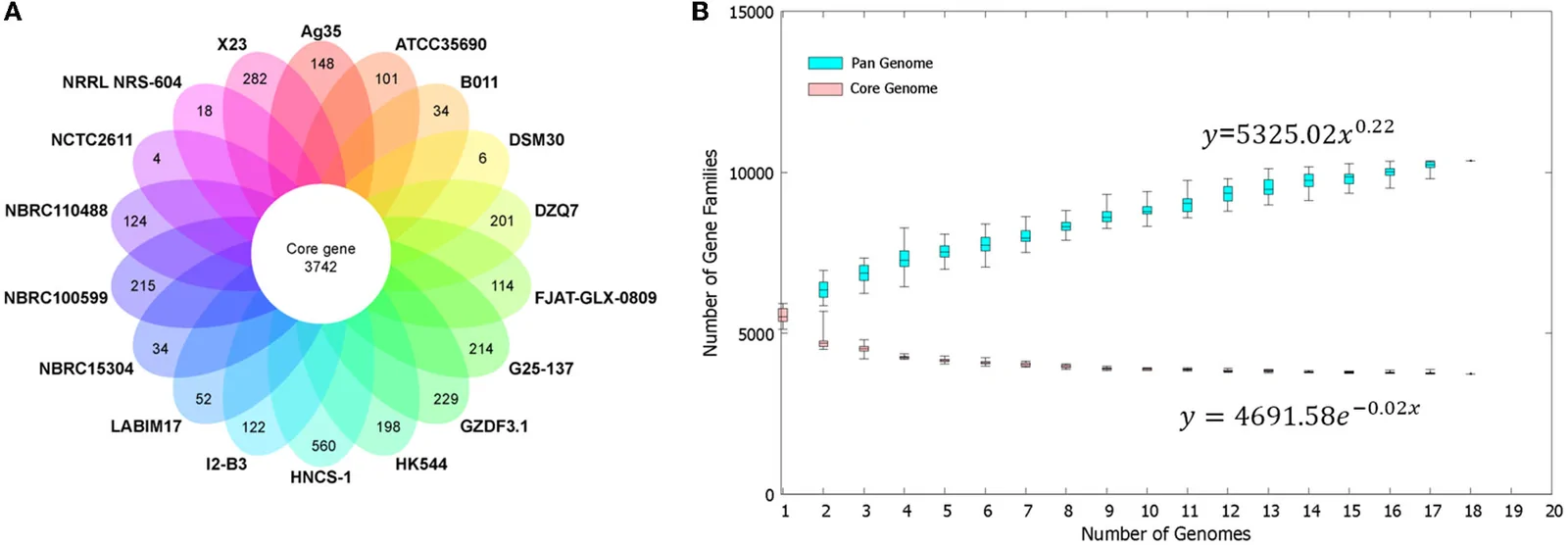
Pan-genome of 18 *Br. brevis* strains. **(A)** The number of unique CDS for each strain of the *Br. brevis* pan-genome. The inner circle shows the core genomes shared between all strains. The specific genes for each strain are indicated in each of the outer circles. **(B)** Curve development of pan (blue color) and core (pink color) genomes. The number of gene families is plotted in function of the genome number.

The BPGA software determined a pan-genome for the strain HNCS-1 and 17 sequenced *Br. brevis* strains by comparing pan-genome analyses of bacterial species by utilizing protein clustering data in the total pan-genome. There were 10,359 CDS in the total pan-genome of the 18 *Br. brevis* strains. Among the 10,359 protein-coding genes, 3,742 core genes represented 36.12% of the genes in the pan-genome of *Br. brevis*. The number of accessory gene families (3,961 genes) was greater than the number of core gene families (3,742 genes). Moreover, *Br. brevis* HNCS-1 contains 1,586 accessory genes. In addition, the strain HNCS-1 also contained the greatest number of distinct transcripts (560 genes) and has greater potential for gene exploration. *Br. brevis* strains NCTC2611 and DSM30 encoded the fewest number of specific strain genes with 4 and 6, respectively (Figure 3A).

The number of *Br. brevis* genomes was plotted against the size of the pan-genome and the core genome. The pan-genome curve exhibited an asymptotic trend, indicating that 18 genomes were inadequate to characterize the complete gene repertoire of *Br. brevis*. According to the curve generated for these 18 genomes based on Heap's Law and least-square fit of exponential regression decay, the number of gene families in the pan-genome increases with the addition of each additional genome (γ = 0.22), indicating that the pan-genome of *Br. brevis* strains is currently open but may be closed soon (Figure 3B).

### Analysis of genes encoding AMPs

**Figure 4:**
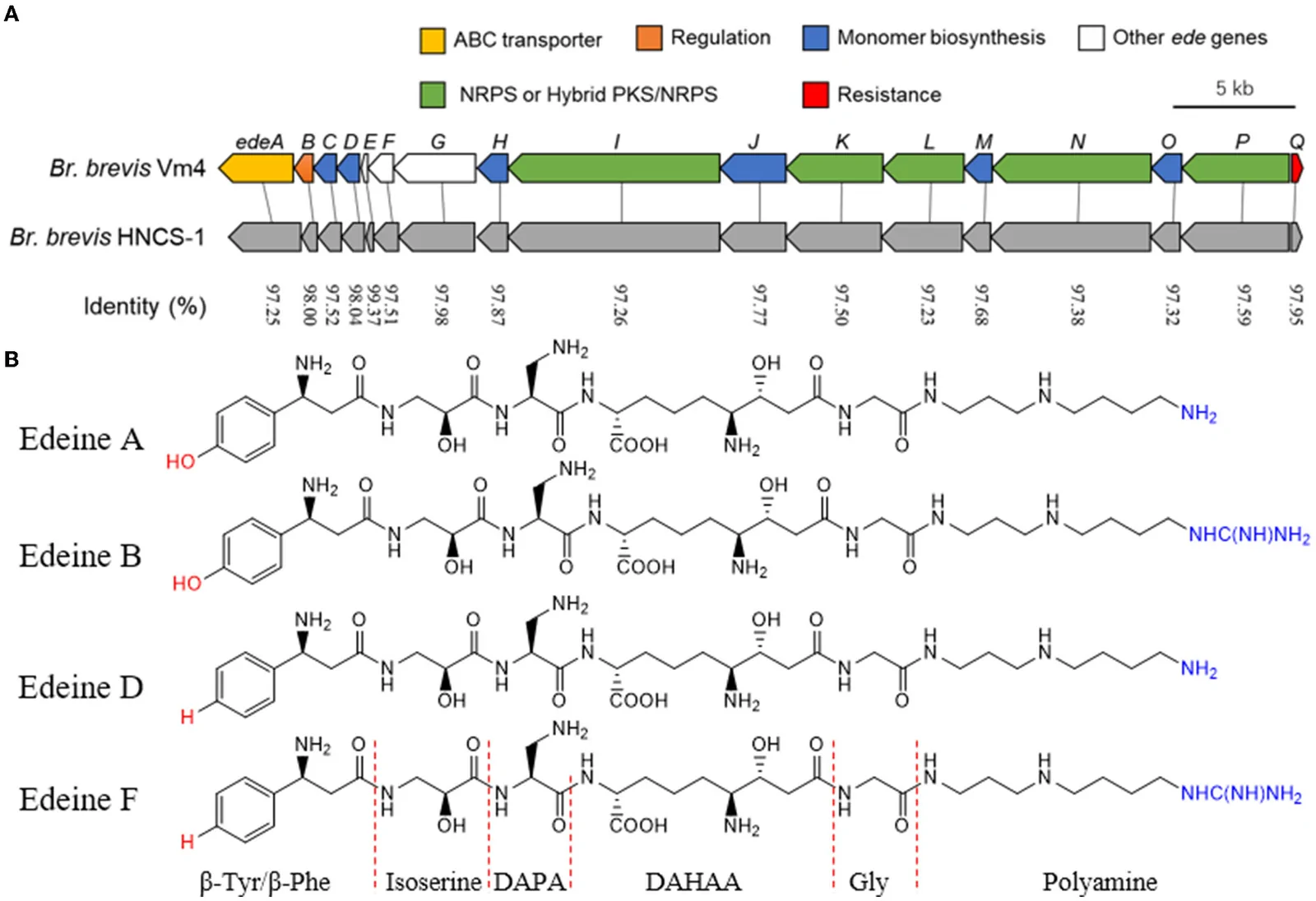
Characteristics of the *ede* BGC gene and structures of edeines. **(A)** Comparison of the *ede* BGC between *Br. brevis* Vm4 and *Br. brevis* HNCS-1. The sequences were compared by NCBI BLAST analysis. The percentage identity is displayed between homologous genes. **(B)** Chemical structure of edeine A, B, D, F. DAPA, 2,3-diaminopropionic acid; DAHAA, 2,6-diamino-7-hydroxyazaleic acid.

It is noteworthy that the cell-free supernatant fermentation of *Br. brevis* inhibited numerous bacterial and fungal diseases. Additionally, the core genome sequence of *Br. brevis* was mined for AMP-encoding genes. Only one gene cluster implicated in edeine NRPS was identified in the core genome after NR annotation. The gene cluster likely contributes significantly to antimicrobial activity. The sequence similarity between the genes and the predicted *ede* BGC of *Br. brevis* Vm4 was high (Westman et al., 2013). The identified *ede* BGC in the *Br. brevis* HNCS-1 genome was structured identically to the *Br. brevis* Vm4 genome. In brief, the *ede* BGC (43.57 kb) in *Br. brevis* HNCS-1 contained 17 open reading frames, designated *ede*A through *ede*Q based on the homologous sequences in *Br. brevis* Vm4 (44.12 kb) (Figure 4A).

Additionally, the genome sequence of *Br. brevis* HNCS-1 was mined by antiSMASH software for the presence of gene-encoding AMPs. A total of 13 putative biosynthetic gene clusters were identified by the HNCS-1 strain genome (Supplementary Figure 7). Four putative gene clusters shared a high degree of similarity (>70% of genes shared similarity) with the petrobactin, tyrocidine, bacillopaline, and gramicidin gene clusters. Three putative gene clusters displayed modest similarity (<30% of genes displayed similarity) to the previously reported zwittermicin A, aurantinin B-D, and pacidamycin (1–7, D) gene clusters. Six putative gene clusters were not conserved in comparison with known clusters.

### Purification and identification of AMPs

The antimicrobial peptide should be edeine, according to the results of genome annotation analysis. Edeines are a class of linear pentapeptides generated by *Brevibacillus*, a soil bacterium. Edeines A, B, D, and F exist as two isomers of α and β; however, only the α isomer possesses remarkable antibiotic properties (Figure 4B).

**Figure 5:**
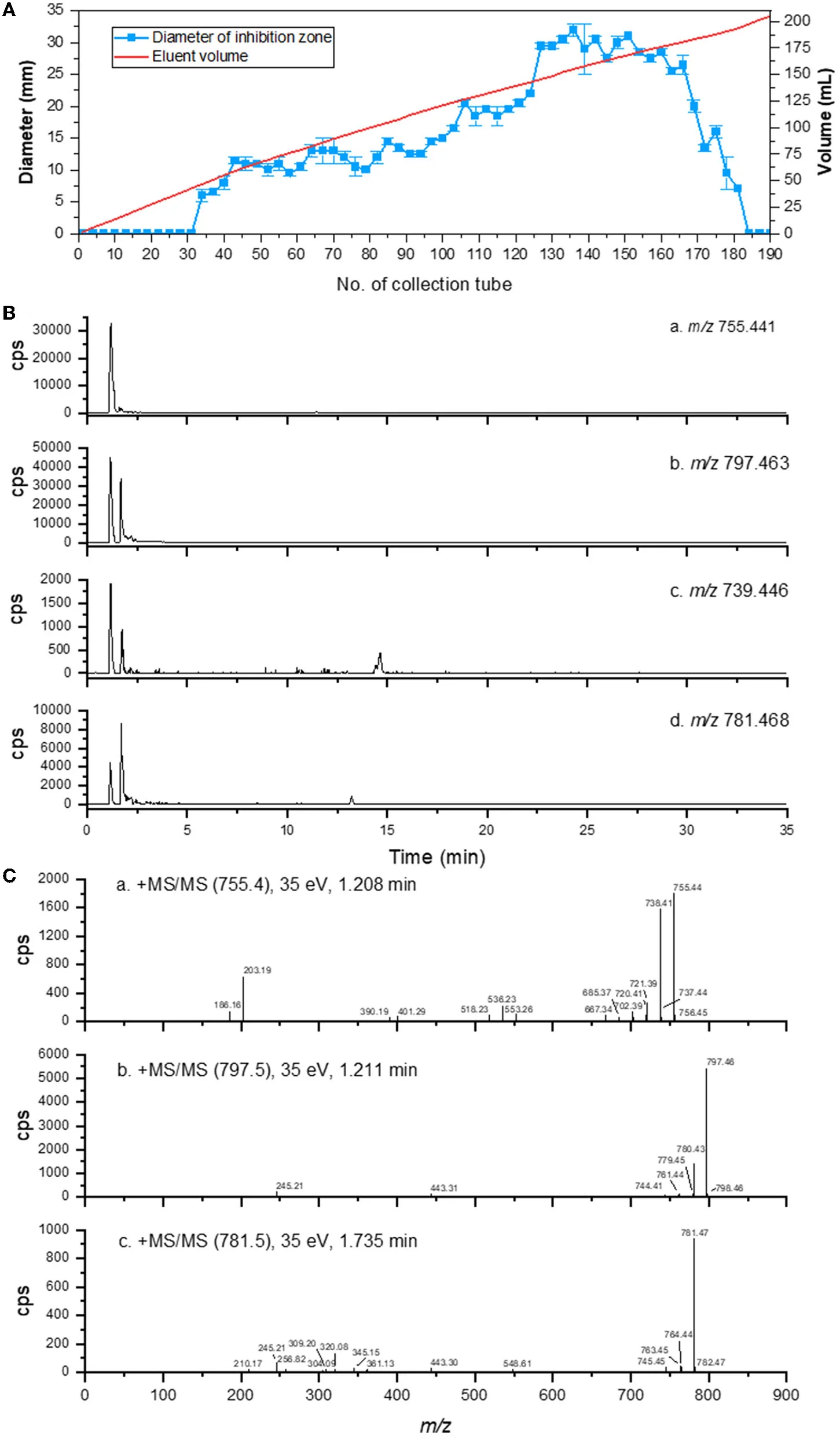
Purification and UHPLC-MS/MS analysis of AMPs from *Brevibacillus brevis* HNCS-1. **(A)** Antimicrobial activity of crude extract of HNCS-1 after elution with H_2_O/CH_3_OH. **(B)** The ion current of *m/z* 755, 797, 739, and 781. **(C)** MS/MS spectra of *m/z* 755, 797, and 781.

The antibacterial potential of eluent extracts containing antibacterial compounds was evaluated. Using an agar well diffusion assay, the antibacterial potential of eluent extracts from distinct collection tubes against *G. theae-sinensis* was determined. With an increase in eluent volume, the bacteriostatic activity initially increased steadily and then decreased dramatically (Figure 5A). The sample with the highest antibacterial activity was analyzed by UPLC-MS/MS for additional confirmation. According to the total ion chromatogram, the majority of the ultraviolet absorption is of short wavelength.

The theoretical molar masses of edeine A (C_33_H_58_N_10_O_10_), edeine B (C_34_H_60_N_12_O_10_), edeine D (C_33_H_58_N_10_O_9_), and edeine F (C_34_H_60_N_12_O_9_) are 754.4337, 796.4555, 738.4388, and 780.4606. Compound 1 (*m/z* 755), Compound 2 (*m/z* 797), Compound 3 (*m/z* 739), and Compound 4 (*m/z* 781) were extracted from the total ion current based on the ion mass spectral (*m/z*). Compound 1 (*m/z* 755) produced a [M+H]^+^ ion peak *m/z* 755.4132 (−1.7 ppm, C_33_H_58_N_10_O_10_) at a retention time of 1.182 min (Figure 5B-a). The electrospray ionization-mass spectrometry results indicated that its fragmentation pattern was identical to that of edeine A, including *m/z* 738, 737, 720, 667, 553, 536, 401, 390, and 203 (Figure 5C-a, Supplementary Figure 8). Compound 2 (*m/z* 797) produced a [M+H]^+^ ion peak *m/z* 797.4612 (−2.6 ppm, C_34_H_60_N_12_O_10_) at a retention time of 1.165 min (Figure 5B-b) with observable signals. The electrospray ionization-mass spectrometry results indicated that its fragmentation pattern was identical to that of edeine B, including *m/z* 780, 779, 761, 443, and 245 (Figure 5C-b, Supplementary Figure 9). Compound 4 (*m/z* 781) produced a [M+H]+ ion peak at *m/z* 781.4667 (−2.2 ppm, C_34_H_60_N_12_O_9_) at the retention time of 1.708 min (Figure 5B-d). The electrospray ionization-mass spectrometry results indicated that its fragmentation pattern was identical to that of edeine F, with fragment ions at *m/z* 764, 763, 746, 745, 722, 443, 287, and 245 (Figure 5C-c, Supplementary Figure 10). To sum up, edeines were a class of highly polar AMPs with limited retention on reverse phase chromatography, which was consistent with the chromatographic retention property of the compounds discussed previously. Compounds 1, 2, and 4 were edeine A, B, and F, respectively. The chromatographic peak area suggested that edeine B showed the highest relative abundance, followed by edeine A, whereas edeine F showed the lowest relative abundance.

In addition, to confirm the existence of secondary metabolites predicted by antiSMASH software, we extracted relevant data from the total ion current based on the theoretical molecular weight of petrobactin, tyrocidine, bacillopaline, and gramicidin, but they were not detected (Supplementary Figures 11–14).

## Discussion

Allelopathy is a pervasive natural phenomenon in ecosystems, promoting and inhibiting the proliferation of diverse organisms. People have always been interested in the rational application of biocontrol's antagonistic effect on agricultural resistance and disease control. Tea plant diseases are difficult to control, resulting in substantial losses in yield and quality. Although chemical fungicides have some effect on the control of tea plant diseases, their misuse has resulted in severe environmental pollution issues. Utilizing biocontrol microorganisms to combat plant maladies is a popular topic of discussion. In certain instances, biocontrol microorganisms have been shown to be as effective as chemical fungicides in preventing plant diseases. Several bacterial species, such as *Bacillus, Brevibacillus*, and *Pseudomonas*, have been commercialized as biological control agents (Zhang et al., 2005; Paterson et al., 2017). Because of their extensive colonization capabilities, spore-forming *Bacillus* and *Brevibacillus* preparations are preferred for the development of commercial products. In this study, we isolated strain HNCS-1 of *Br. brevis* from tea garden soil that exhibits broad-spectrum resistance to five fungal diseases of tea plants. The HNCS-1 strain is a beneficial microorganism with prospective biotechnological application potential.

The HNCS-1 strain and 17 *Br. brevis* isolates from NCBI were subjected to a phylogenetic analysis using orthologous genes organized into four clusters. However, the strains of the same cluster originate from diverse habitats, indicating that *Br. brevis* is highly adaptable to its environment and can populate a variety of ecological niches. We examined the pan-genome of *Br. brevis* species. The results indicate that the pan-genome of *Br. brevis* is open and theoretically infinite, indicating that *Br. brevis* species tend to acquire new genes to improve adaptability. Bacteria could modify their genetic material to adapt to varying environmental conditions, resulting in increased niche diversity and larger pan-genomes (Konstantinidis and Tiedje, 2004). The HNCS-1 strain has more protein-coding genes (6,342) than other *Br. brevis* strains, making it more environmentally adaptable.

In addition, the traditional separation methods of antimicrobial substances have limitations. According to previous studies, this study tried to extract antimicrobial substances through the organic solvent extraction method, hydrochloric acid precipitation method, and ammonium sulfate precipitation method but never succeeded. Genome sequencing analysis and pan gene analysis open a new door for the identification of antimicrobial substances, which can quickly and accurately target antimicrobial substances and lay a theoretical foundation for the separation and application of antimicrobial substances.

A comparison of *Br. brevis* HNCS-1 proteins with those of other *Br. brevis* strains revealed 3,742 core genes. There is only one NRPS gene cluster annotated as edeine in the core genes. It is demonstrated that edeine is the antibacterial peptide at the center of *Br. brevis*. After a blast comparison, it was determined that the gene cluster of the HNCS-1 strain was extremely similar to the *Br. brevis* Vm4 gene cluster for edeine NRPS (identity >97%). To validate the accuracy of comprehensive genomic analysis, we used the UHPLC-MS/MS technique to identify edeine A, B, and F from the crude extract of the HNCS-1 strain, with edeine B and edeine A serving as the principal AMPs. The main species of edeine in the HNCS-1 strain differ from *Br. brevis* X23 (Du et al., 2022), which may be owing to the evolutionary tree's distant genetic relationship.

Edeine, as an AMP of the core genes of *Br. brevis*, should be abundant in the organism's secondary metabolites. Edeine was extracted from *Br. brevis* Vm4 and X23 by resin adsorption, respectively (Westman et al., 2013; Liu et al., 2022). However, ethylparaben from *Br. brevis* FJAT-0809-GLX was extracted by solvent extraction (Che et al., 2015), tostadin from *Br. brevis* XDH was extracted by ammonium sulfate precipitation (Song et al., 2012), siderophore from *Br. brevis GZDF3* was extracted by ethanol precipitation (Sheng et al., 2020), and surfactin from *Br. brevis* KN8(2) was extracted by hydrochloric acid precipitation (Krishnan et al., 2019). The inability to extract edeine from the secondary metabolites of *Br. brevis* may have been caused by a mismatch in extraction techniques. Due to the ability of *Br. brevis* to acquire new genes from the environment, its antimicrobial peptides are diverse. The pan-genome analysis identified 560 HNCS-1 strain-specific genes. The unique genomes of the HNCS-1 strain require additional study. Additionally, genetic engineering is an excellent research direction. For instance, using *in situ* promoter engineering, the production of edeine in *Br. brevis* X23 was increased. *Br. brevis* HNCS-1 requires additional research, including the one strain many compounds approach (OSMAC) and genetic modification, to investigate novel compounds and yield enhancement.

## Data availability statement

The data presented in the study are deposited in the GenBank repository, accession number CP128411.

## Author contributions

CX, LZ, and XQ conceived and planned the experiments, funding acquisition, and revisions. WY and HY were involved in data and bioinformatics analyses, conducted experiments, and wrote the manuscript. XB and MH contributed to the interpretation of the results. QB and ZZ contributed to sample preparation. All authors contributed to the article and approved the submitted version.
