# Correction: *Brevibacillus brevis* HNCS-1: a biocontrol bacterium against tea plant diseases

**DOI:** 10.3389/fmicb.2026.1958077

**Published:** 2026-09-10

**Authors:** Wenbo Yang, Hui Yang, Xiaocun Bao, Mehboob Hussain, Qiang Bao, Zexuan Zeng, Chun Xiao, Lingyun Zhou, Xiaoping Qin

**Affiliations:** 1 College of Plant Protection, Yunnan Agricultural University, Kunming, China; 2 Tea Research Institute, Hunan Academy of Agricultural Sciences, Changsha, China

**Keywords:** *Brevibacillus brevis*, antagonistic activity, genome annotation, pan-genome analysis, antimicrobial peptides, edeine

In the original publication, the scientific name of the tea white scab pathogen was incorrectly written as “*Elsinoe leucospira*”. This has been corrected to “*Elsinoe leucospila*” throughout the text.

The fermentation temperature was incorrectly reported in the Section **Materials and methods**, subsection *Antibiotics activity of* Brevibacillus brevis *HNCS-1*, paragraph 1. This originally read: “*Br. brevis* HNCS-1 was inoculated into 500 ml Erlenmeyer flasks containing 200 ml of the LB liquid medium and cultivated at 37 °C and 200 rpm for 12 h. Then, 10 ml of the seed cultures were transferred to 500 ml Erlenmeyer flasks containing the NB medium (10 g peptone, 3 g beef extract, 5g NaCl, 1,000 ml H_2_O, and pH 7.2 ± 0.2) and cultivated at 37 °C and 180 rpm for 3 days.” The text has been updated to read:

“*Br. brevis* HNCS-1 was inoculated into 500 ml Erlenmeyer flasks containing 200 ml of the LB liquid medium and cultivated at 37 °C and 200 rpm for 12 h. Then, 10 ml of the seed cultures were transferred to 500 ml Erlenmeyer flasks containing the NB medium (10 g peptone, 3 g beef extract, 5 g NaCl, 1,000 ml H_2_O, and pH 7.2 ± 0.2) and cultivated at 26 °C and 180 rpm for 3 days.”

The pathogen incubation description contained an incomplete pathogen list and an incorrectly spelled scientific name in the Section **Materials and methods**, subsection *Antibiotics activity of* Brevibacillus brevis *HNCS-1*, paragraph 2. This originally read: “Plates of *P. theaefolia, Fusarium* sp., *E. leucospira*, and *C. theae* were incubated at 26 °C for 12 days, 11 days, 22 days, 22 days, and 7 days, respectively.” This has been updated to read:

“Plates of *P. theaefolia, Fusarium* sp., *E. leucospila, C. theae*, and *G. theae-sinensis* were incubated at 26 °C for 12 days, 11 days, 22 days, 22 days, and 7 days, respectively.”

The description of Compound 4 in the Section **Results**, subsection *Purification and identification of AMPs*, paragraph 3, contained an incorrect compound annotation and MS/MS fragmentation description. This originally read: “Compound 4 (*m/z* 781) produced a [M+H]^+^ ion peak *m/z* 781.4667 (−2.2 ppm, C_34_H_60_N_12_O_9_) at the retention time of 1.708 min (Figure 5B-d). The electrospray ionization-mass spectrometry results indicated that its fragmentation pattern was identical to that of edeine B, including *m/z* 780, 779, 761, 443, and 245 (Figure 5C-c, Supplementary Figure 10).” This has been updated to read:

“Compound 4 (*m/z* 781) produced a [M+H]+ ion peak at *m/z* 781.4667 (−2.2 ppm, C_34_H_60_N_12_O_9_) at the retention time of 1.708 min (Figure 5B-d). The electrospray ionization-mass spectrometry results indicated that its fragmentation pattern was identical to that of edeine F, with fragment ions at *m/z* 764, 763, 746, 745, 722, 443, 287, and 245 (Figure 5C-c, Supplementary Figure 10).”

The description of the relative abundance of edeine analogues was incorrect in the Section **Results**, subsection *Purification and identification of AMPs*, paragraph 3. This originally read: “The chromatographic peak area suggested that edeine B had the maximum concentration, followed by edeine B and F had the lowest concentration.”. This has been updated to read:

“The chromatographic peak area suggested that edeine B showed the highest relative abundance, followed by edeine A, whereas edeine F showed the lowest relative abundance.”

Several small typographical errors have also been identified in the published article.

In the **Abstract**, “showd” has been corrected to “showed” in the sentence “And UHPLC-MS/MS detection results showd that edeine B and edeine A were the principal antibacterial peptides in the HNCS-1 strain.”

In the **Introduction**, paragraph 2: “eptide” has been corrected to read “peptide” in the sentence “Currently, the preponderance of *Brevibacillus* AMPs are produced by non-ribosomal eptide synthetases.”.

In the Section **Results**, subsection *General genome description of* Brevibacillus brevis *HNCS-1*, paragraph 2, “Pfm” has been corrected to “Pfam” in the sentence “Moreover, 4,808, 2,906, 1,190, 173, and 6 genes, respectively, were annotated in Pfm, PHI, VFDB, CAZyme, and CARD (Supplementary Figures 5, 6).”.

The original version of this article has been updated.

