# Supplementary material for: Brevibacillus brevis HNCS-1: a biocontrol bacterium against tea plant diseases
Source: Front Microbiol. 2023 Sep 13;14:1198747. doi: 10.3389/fmicb.2023.1198747 (PMC10534016; doi:10.3389/fmicb.2023.1198747)
Supplement: Supplementary file 1 [file Data_Sheet_1.docx]

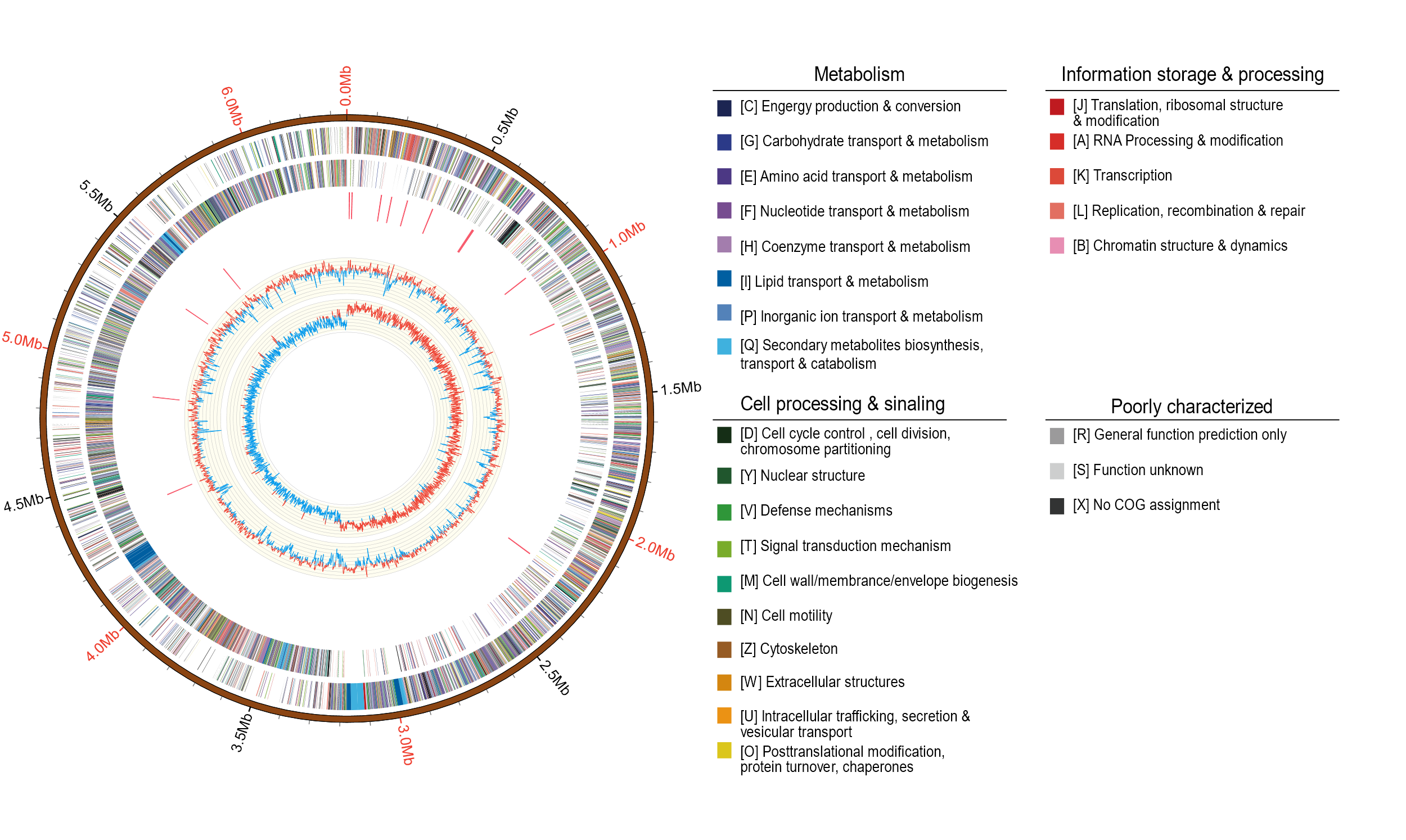


Figure S1 The genome map of *Brevibacillus brevis* HNCS-1. Rings from the outermost to the center: a. scale marks of the genome; b. protein-coding genes on the forward strand; c. protein-coding genes on the reverse strand; d. tRNA (black) and rRNA (red) genes on the forward strand; e. GC content; f. GC skew. Protein-coding genes were color coded according to their COG categories.


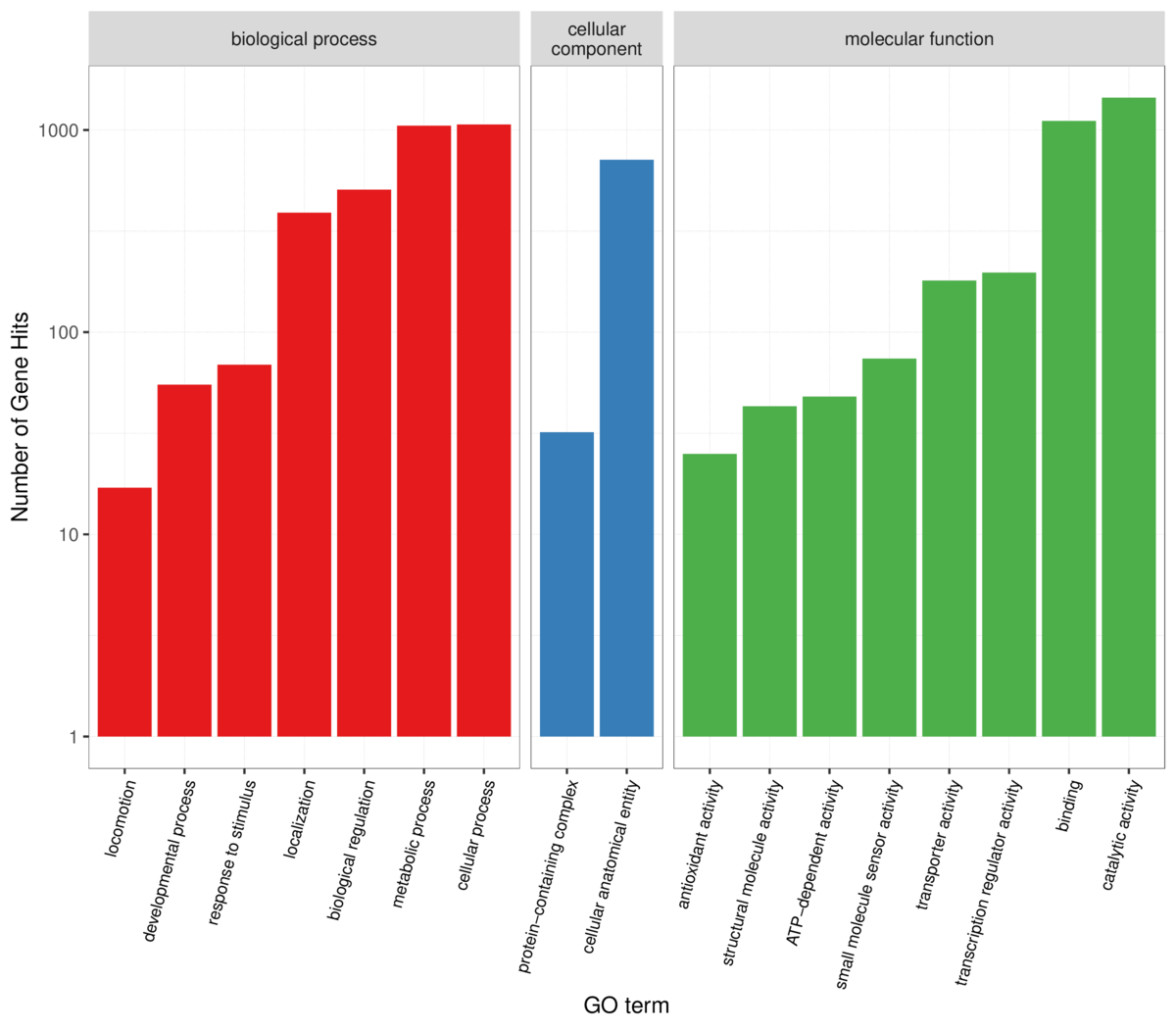


Figure S2 GO-based functional classification of genes located on HNCS-1 genome


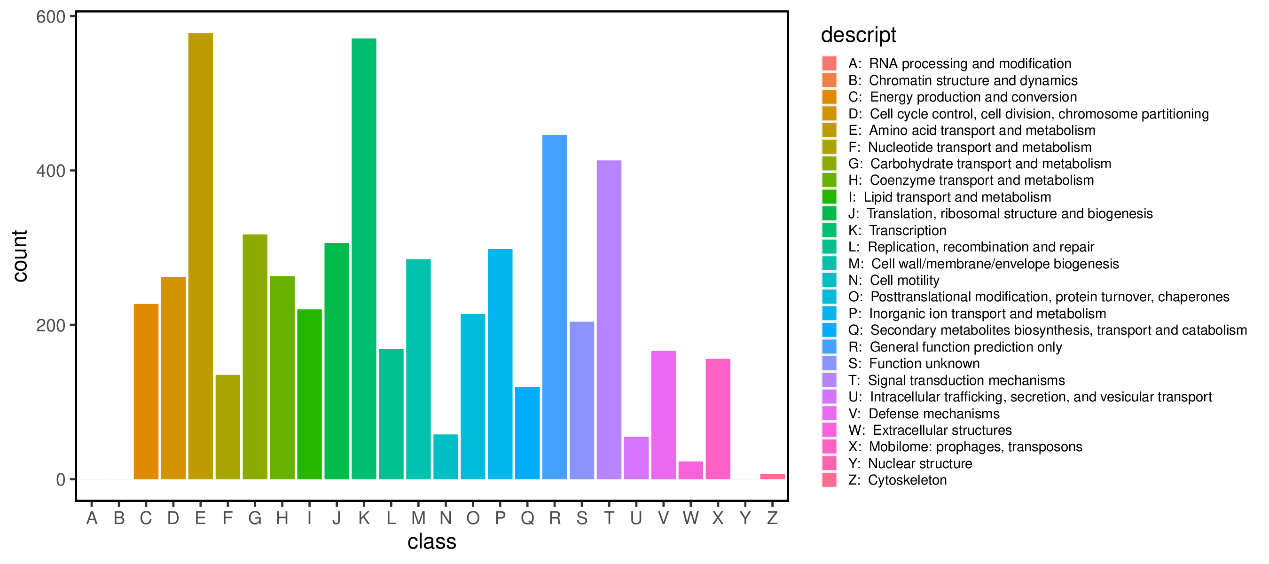


Figure S3 COG-based functional classification of genes located on HNCS-1 genome


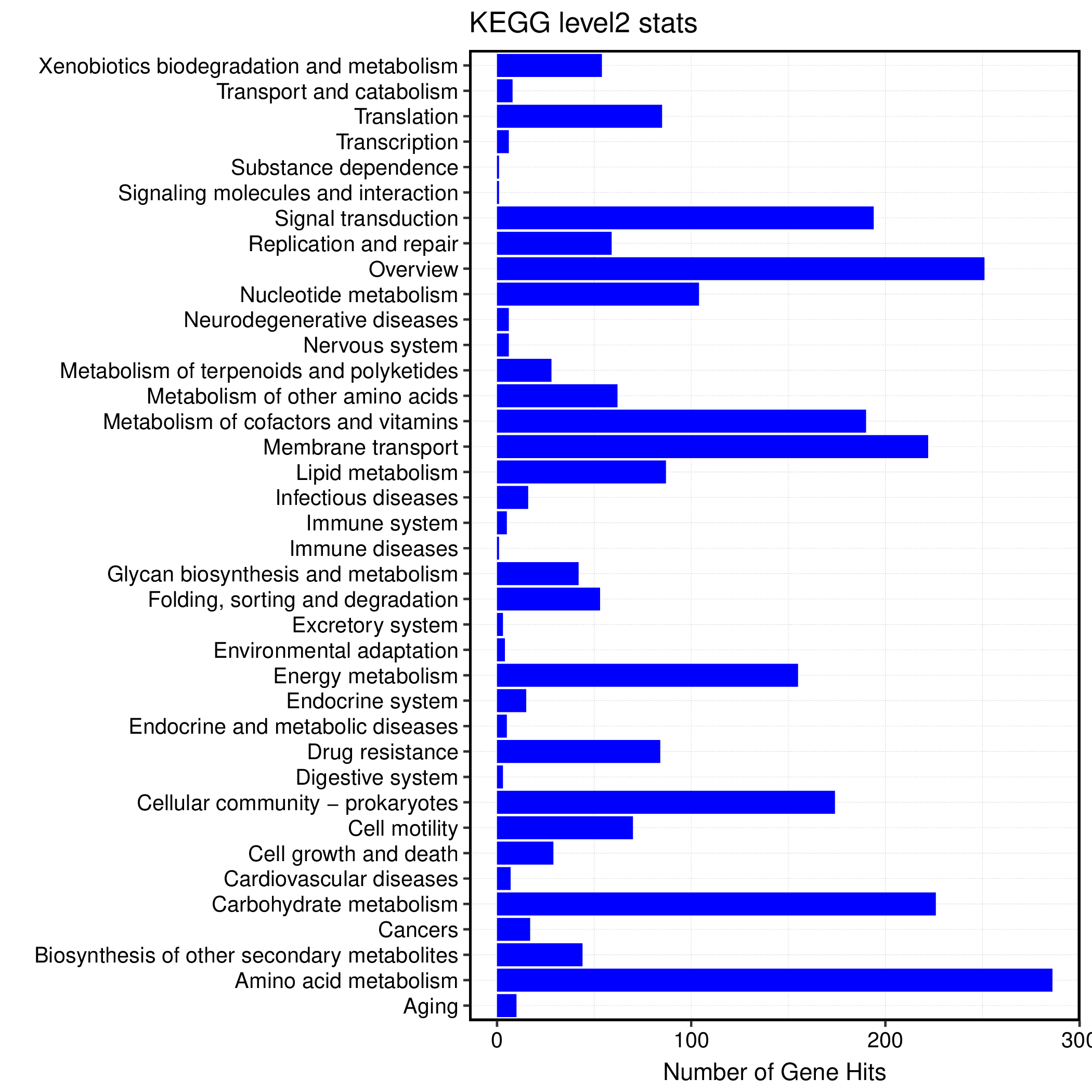


Figure S4 KEGG-based functional classification of genes located on HNCS-1 genome


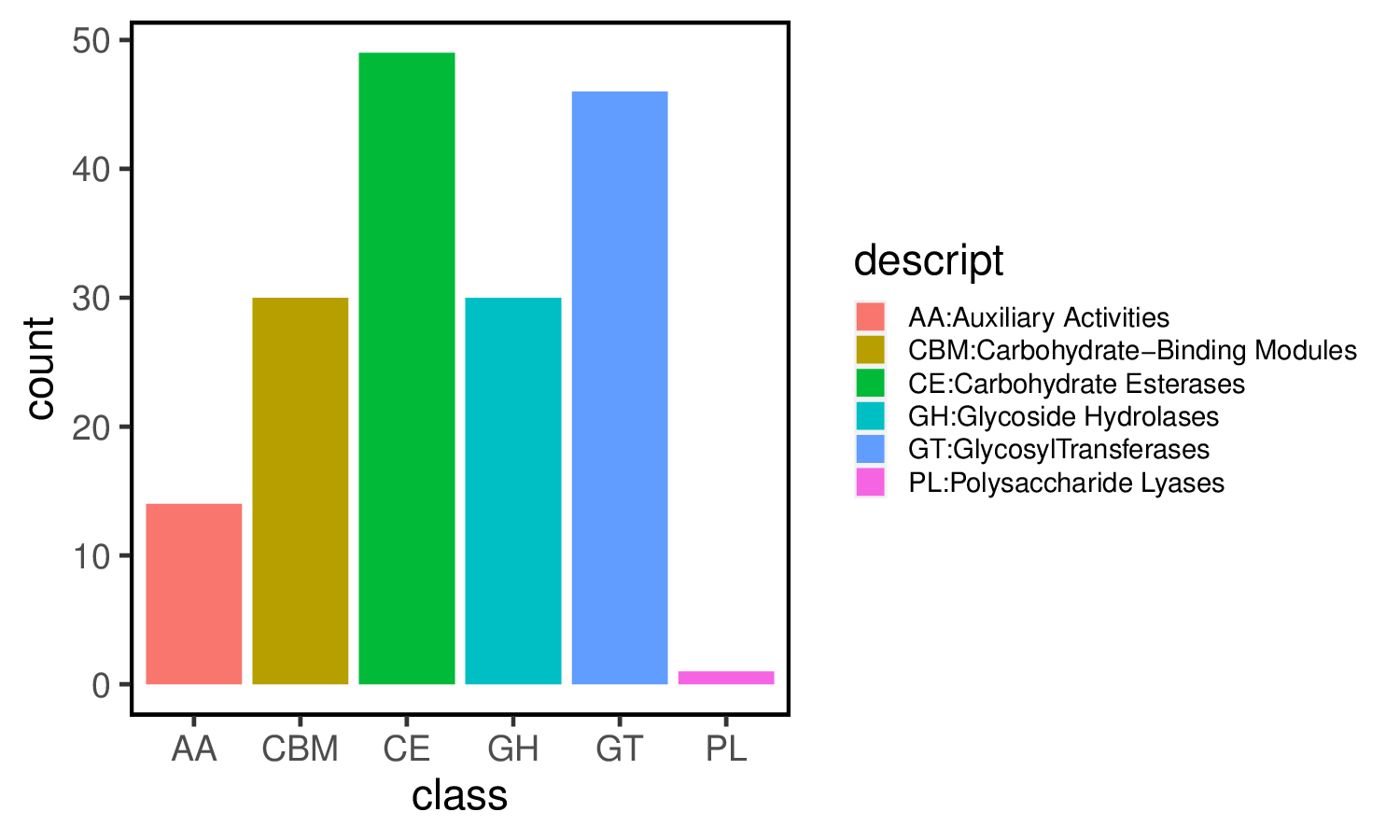


Figure S5 CAZyme-based functional classification of genes located on HNCS-1 genome


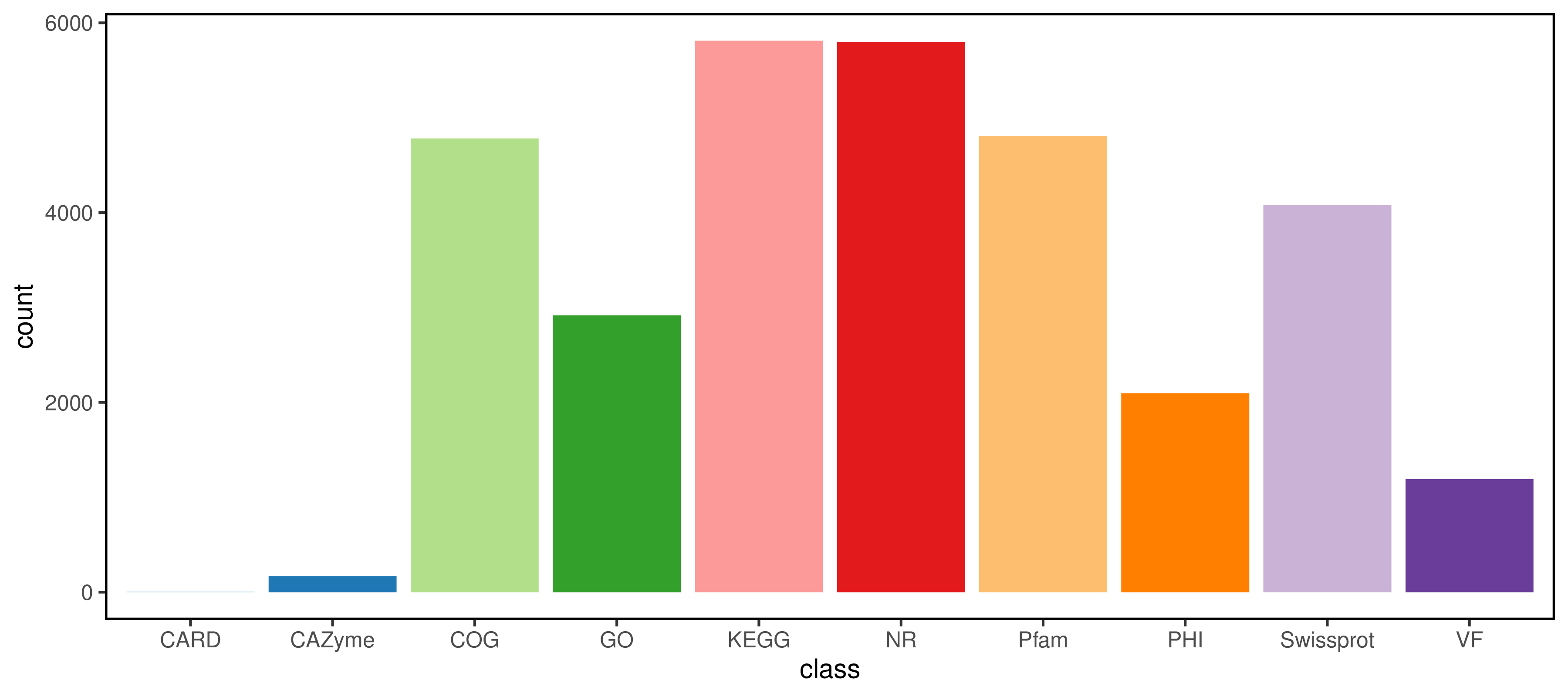


Figure S6 Number of gene annotations of HNCS-1 genome in 10 databases


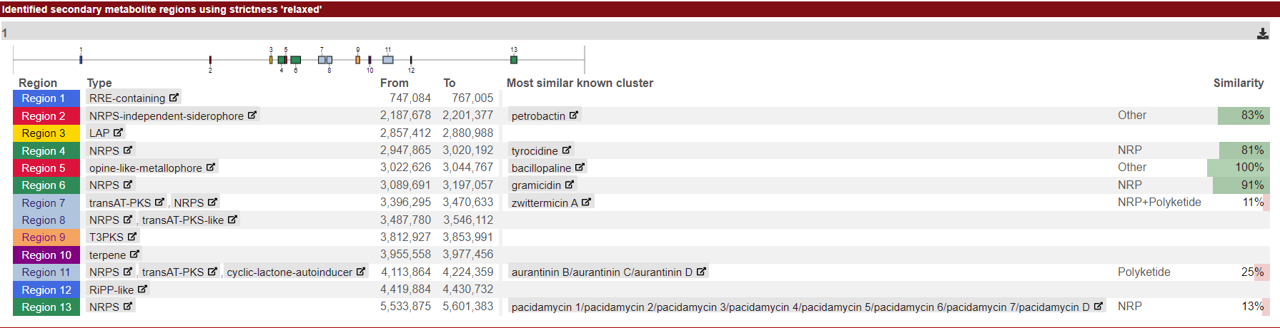


Figure S7 putative biosynthetic gene clusters of HNCS-1 genome

Figure S8 The mass spectral fragmentation of edeine A.

Figure S9 The mass spectral fragmentation of edeine B.

Figure S10 The mass spectral fragmentation of edeine F.


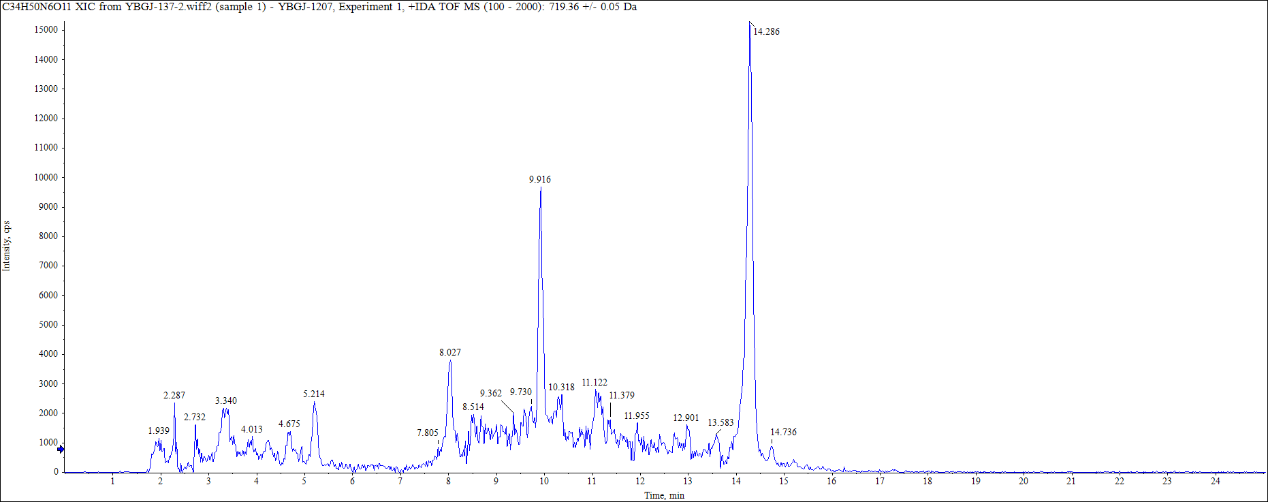


Figure S11 The iron current of petrobactin (C_34_H_50_N_6_O, *m/z* 719.36)


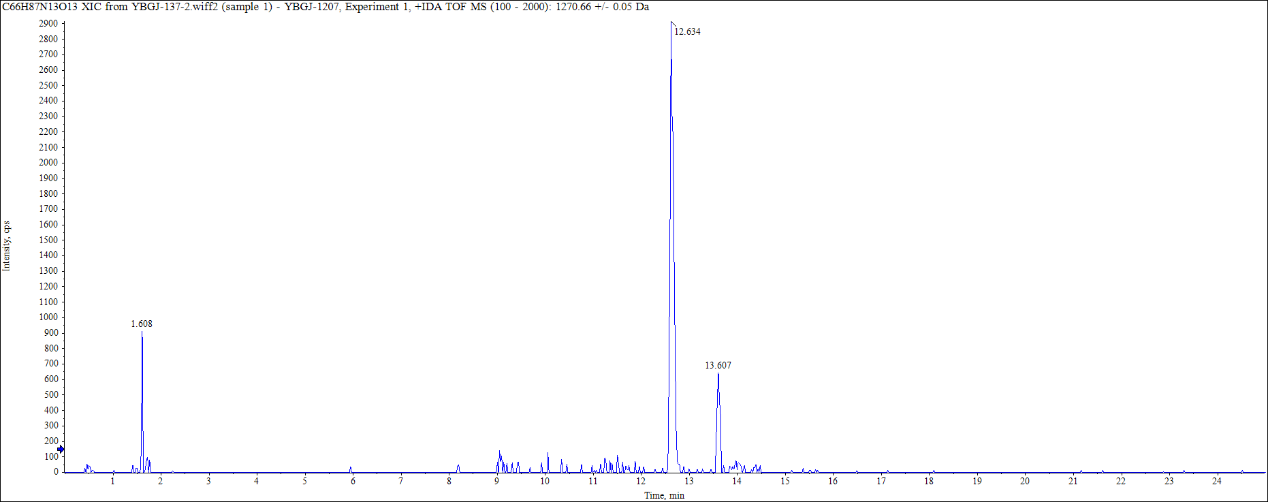


Figure S12 The iron current of tyrocidine (C_66_H_87_N_13_O_13_, *m/z* 1270.66)


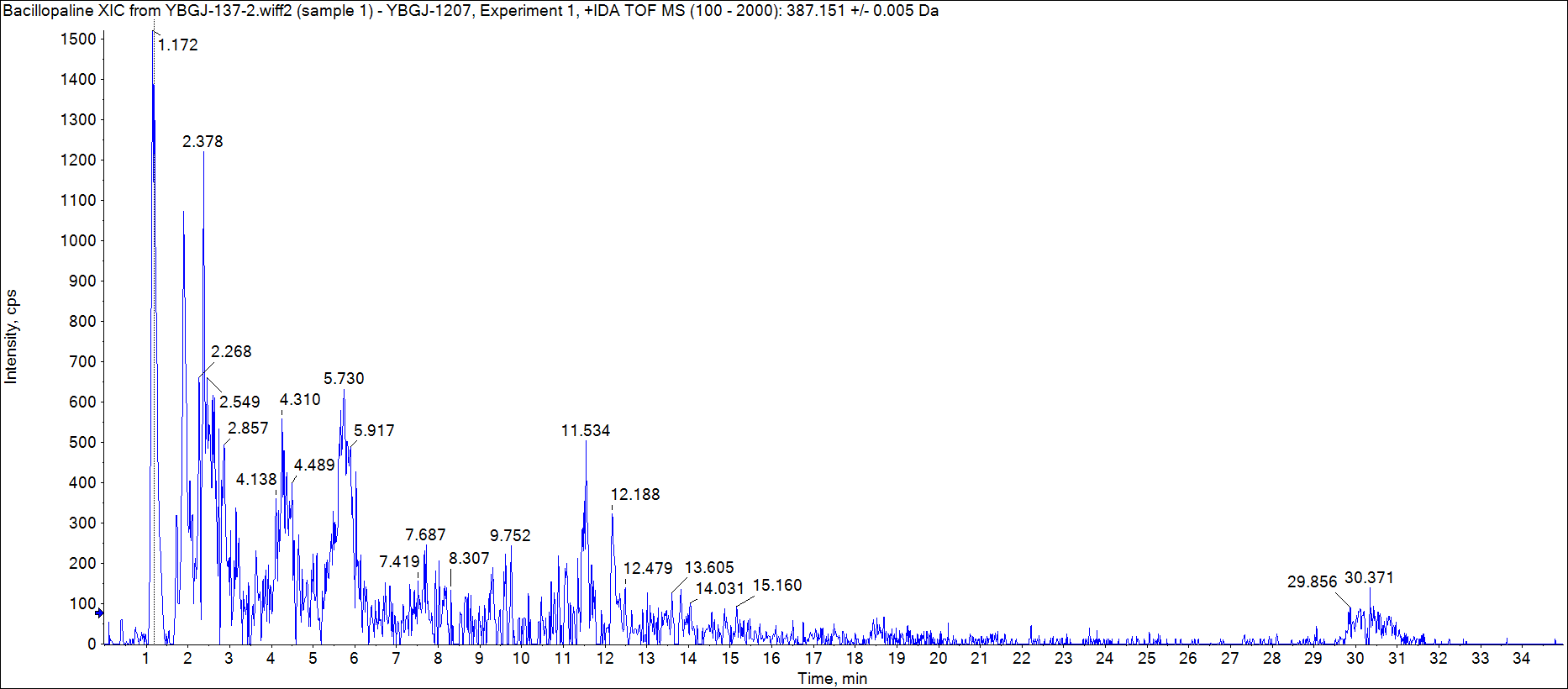


Figure S13 The iron current of bacillopaline (C_15_H_22_N_4_O_8_, *m/z* 387.15)


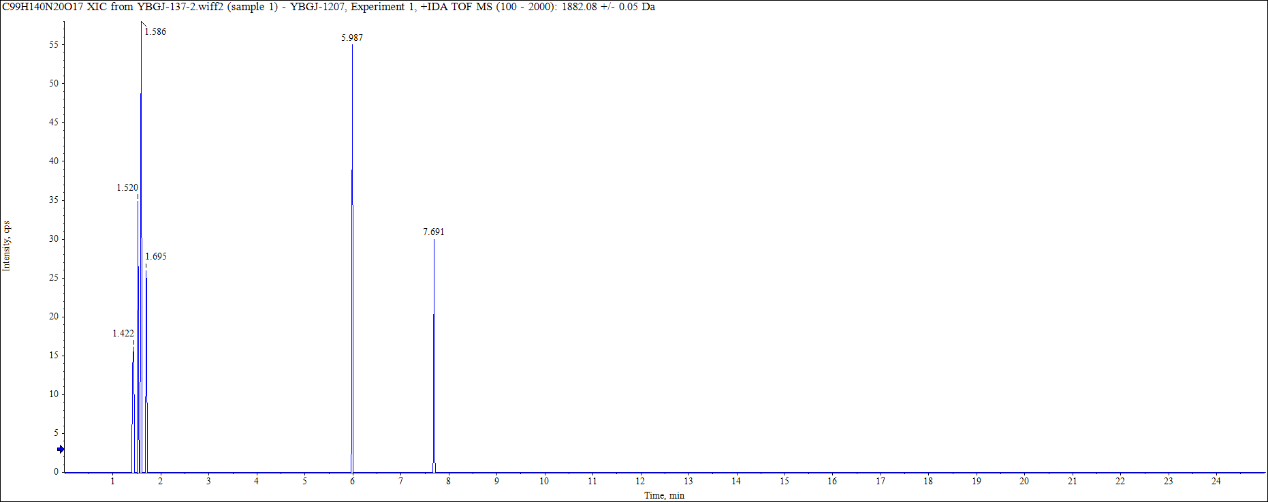


Figure S14 The iron current of gramicidin (C_99_H_140_N_20_O_17_, *m/z* 1882.08)
